# Epirubicin/paclitaxel/etoposide in extensive-stage small-cell lung cancer: a phase I–II study

**DOI:** 10.1038/sj.bjc.6603074

**Published:** 2006-04-04

**Authors:** C Tibaldi, T Prochilo, F Russo, M C Pennucci, A Del Freo, F Innocenti, A Fabbri, A Falcone, P F Conte, E Baldini

**Affiliations:** 1 Division of Medical Oncology, Civil Hospital, Livorno, Italy; 2 Division of Medical Oncology, S Chiara Hospital, Pisa, Italy; 3 Division of Medical Oncology, Civil Hospital, Carrara, Italy; 4 Division of Pneumology, Civil Hospital, Pistoia, Italy; 5 Department of Oncology, Transplants and Advanced Technologies, University of Pisa, Italy; 6 Department of Oncology, University of Modena, Italy

**Keywords:** epirubicin, paclitaxel, etoposide, SCLC

## Abstract

The aim of this study was to evaluate feasibility and toxicity of escalating doses of epirubicin and paclitaxel plus fixed dose of etoposide and to define the activity of the triplet in extensive disease small-cell lung cancer. Thirteen patients entered the phase I study: the maximum tolerated doses were epirubicin (EpiDX) 90 mg m^−2^ and paclitaxel (P) 175 mg m^−2^ with febrile neutropenia as dose-limiting toxicity. The recommended schedule for this regimen for the phase II study was EpiDX 75 mg m^−2^, P 175 mg m^−2^, etoposide (E) 100 mg m^−2^ intravenous (fixed dose) days 1–3 with courses repeated every 21 days. The prophylactic use of colony-stimulating factors (CSFs) was not allowed. Twenty patients entered the phase II trial: median age was 61 years (range 50–70), median Eastern Cooperative Oncology Group performance status 0 (0–2); nine patients had visceral disease and 17 had more than two metastatic sites. A total of 100 courses were administered with a median of 5 (range 1–6) per patients. Main toxicity (NCI-CTC) was myelosuppression: neutropenia grades 3 and 4 in 16 and 35% of the courses, respectively. Seven episodes of febrile neutropenia were documented and one patient required hospital admission. Nonhaematological toxicity was moderate. Seven out of 19 evaluable patients achieved a complete response (37%), nine patients (47.3%) a partial response with an overall response rate of 84.2% ((95% confidence interval=60.4–96.6)). In this poor prognostic population of patients the triplet epirubicin/paclitaxel/etoposide showed high antitumour activity with mild nonhaematological side effects. The use of CSFs should be able to improve the haematological profile.

Small-cell lung cancer (SCLC) accounts for about 20% of primary lung cancers and approximately 60% of SCLC patients have extensive disease (ED) at diagnosis (Coleman *et al*, 1993). In this disease, combination regimens are clearly superior to single-agent ones and they represent the standard of care for patients with good performance status (PS). However, despite the initial high sensitivity to treatment, in most cases the disease will ultimately relapse: this clinical behaviour stresses the need to introduce new agents and/or new combinations in the management of this poor prognosis population of patients.

Anthracyclines are highly active against SCLC and they are included in some of the most widely used combination regimens such as cyclophosphamide, doxorubicin, vincristine (CAV) and cyclophosphamide, doxorubicin, etoposide (CDE). Cisplatin/etoposide (PE) is the reference regimen for the treatment of SCLC in the United States, while CDE represented one of the reference regimens in European countries.

So far, intravenous etoposide has been the cornerstone of SCLC chemotherapy and its activity is clearly schedule dependent. Slevin *et al* compared a 1-day intravenous (i.v.) injection of etoposide (500 mg m^−2^) to the same dose fractionated over 5 days, every 3 weeks; response rates and survival significantly favoured the 5-day schedule (Slevin *et al*, 1989). Activity, efficacy and safety of prolonged oral etoposide have also been evaluated in SCLC: two randomised phase III trials compared single-agent oral etoposide to conventional polichemotherapy in elderly or frail (WHO PS ⩾2) patients: the results of these studies cast serious doubts on the role of oral etoposide in this histology (Medical Research Council Lung Cancer Working Party, 1996; Souhami *et al*, 1997).

Among the last generation drugs tested in this disease, paclitaxel was very promising. This drug showed encouraging single-agent activity as second-line treatment: in patients with ED pretreated with CDE as first-line combination, single-agent paclitaxel showed an overall response rate of 29%, which is at the upper level of activity for any single agent in this setting (Smit *et al*, 1998). An interesting activity was also observed in untreated patients; in the Eastern Cooperative Oncology Group (ECOG) phase II study a 34% remission rate and a 43-week median survival were observed (Ettinger *et al*, 1995); the North Central Cancer Treatment Group (NCCTG) also reported a 53% response rate in 43 untreated patients (Kirschling *et al*, 1999). These phase II studies, evaluating the activity of paclitaxel in ED–SCLC, provided the basis for inclusion of this drug in polichemotherapy regimens.

On these grounds we performed a phase I/II study to evaluate the maximum tolerated dose (MTD) of paclitaxel and epirubicin in combination with a fixed dose of i.v. etoposide as first-line therapy in ED–SCLC, and to evaluate the toxicity and activity of the triplet, particularly in terms of complete response.

## PATIENTS AND METHODS

### Eligibility criteria

The phase I–II study was carried out in chemotherapy-naïve patients with histologically proven ED–SCLC, ECOG PS ⩽2 and aged 70 years or younger. Adequate pretreatment haematological (WBC count ⩽4000 *μ*l^−1^, haemoglobin ⩾10 g dl^−1^, platelet count ⩾100 000 *μ*l^−1^), hepatic (bilirubin at the normal level) and renal (creatinine at the normal level) functions were required. Left ventricular ejection fraction (LVEF) >50% at bidimensional ultrasonography or MUGA scan was mandatory. Patients with brain metastases were eligible. Patients were excluded if they had a positive history of any other type of cancer, with the exception of radically resected *in situ* cervical cancer and nonmelanoma skin cancer, congestive heart failure or other serious medical or psychiatric illnesses. Written informed consent was obtained from each patient according to local institution policies. The protocol was approved by the ethical committees of participating institutions and was conducted according to Good Clinical Practice guidelines and the Helsinki declaration of the World Medical Association. The study was supported by Bristol-Myers Italy.

### Staging and follow-up procedures

Pretreatment evaluation was performed within 4 weeks before the beginning of treatment and consisted of: clinical history and physical examination, complete blood cell counts with differential and biochemistry, bronchoscopy, chest X-ray, computed tomography scan of the chest and abdomen. Other types of organ-specific scanning were optional but recommended in cases of symptoms or biochemical abnormalities. During the study, patients underwent clinical examination and routine biochemistry before day 1 of each course, while blood cell count was performed weekly.

Restaging was planned every three courses of chemotherapy: all target lesions were reassessed with the same method used on study entry according to WHO criteria (Miller *et al*, 1981). Patients were considered evaluable for response if they had received at least three courses of chemotherapy. All responses were centrally reviewed and had to be confirmed 28 days or more after the initial documentation of response.

Toxicities were assessed using the National Cancer Institute common toxicity criteria, version 2.0 (NCI-CTC) (National Cancer Institute, 1999) and reported as the worst toxicity observed per course at any time in the trial.

### Treatment plan

Patients entered the phase I part of this study in triplets. Starting doses were paclitaxel 155 mg m^−2^ (as a 3-h infusion) on Day 1, epirubicin 60 mg m^−2^ (as a slow bolus injection immediately before paclitaxel) on day 1, etoposide 100 mg m^−2^ i.v. (1-h infusion immediately after Paclitaxel) on days 1–3. Premedication for paclitaxel consisted of 20 mg desamethasone by i.v. infusion 30 min before treatment; orphenadrine 40 mg i.m. plus i.v. cimetidine 300 mg 1 h before the start of treatment. The doses of epirubicin and paclitaxel were escalated in consecutive triplets of patients until dose-limiting toxicity (Table 1). No intrapatient escalation was allowed. Chemotherapy was administered on an outpatient basis every 3 weeks for a maximum of six courses.

Dose-limiting toxicities (DLT) were defined as the occurrence of one of the following: absolute neutrophil count (ANC) ⩽500 *μ*l^−1^ lasting more than 7 days or ANC⩽100 *μ*l^−1^ for 3 days (72 h); febrile neutropenia; any grade ⩾3 (NCI-CTC) nonhaematological toxicity except for alopecia. When a DLT was observed in one out of three patients, three more patients were added at the same dose level. If an additional patient experienced a DLT, no further dose escalation was allowed and that dose level was considered as the MTD. The dose level immediately below MTD was the recommended dose for the phase II study.

In the case of grade 4 neutropenia ciprofloxacin 500 mg were administered orally twice a day and fluconazole 100 mg daily until ANC>1000 *μ*l. The prophylactic use of colony-stimulating factors (CSFs) was not allowed but suggested in case of febrile neutropenia. Patients were admitted to the hospital when febrile neutropenia lasted more than 72 h despite the use of adequate orally administered antibiotics, antimicotics and CSFs.

### Statistical analysis

Having the complete response rate as primary end point, Simon's optimal two-stage design for phase II trials was used to calculate the sample size: with an *α* error of 0.05 and a *β* error of 0.10, P0 (clinically uninteresting true complete response rate) and P1 (sufficiently promising true complete response rate) were set at 10 and 30%, respectively. In the first step, 18 patients had to be included: if ⩽2 complete responses were obtained, accrual was stopped, otherwise 18 more patients had to be registered. Drug combination was considered of interest if ⩾7 complete responses were observed out of 36 evaluable patients. Time to progression (TTP) was calculated from the date of registration to the date of clinical and/or radiological evidence of progression or death, whichever occurred first. Survival was calculated from registration to death or last follow-up. Survival and TTP were estimated using the Kaplan–Meier method (Kaplan and Meier, 1993). Data were analysed using SPSS/PC+11.5 statistical software.

## RESULTS

A total of 33 patients entered the phase I/II trial. Accrual into this study was stopped as soon as the number of complete responses required by the statistical design was achieved. Characteristics of patients were as follows: median age 62 years (range 50–70), median ECOG PS 0 (range 0–2); the majority of patients had 2 or more metastatic sites. Demographics are summarized in Table 2.

### Phase I study

Thirteen patients were registered: 18 courses were administered at dose level A (three patients), 18 at dose level B (three patients) and 32 at level C (seven patients); median number of courses administered per patient 6 (range 1–6). The major toxicity associated with this regimen was myelosuppression: a grade 4 neutropenia was observed in 11% of the courses in group B and in 47% of the courses in group C; no grade 4 neutropenia was observed in group A. No grade 4 thrombocytopenia or anaemia were observed. The MTD was found at dose level C (EpiDX, 90 mg m^−2^; P, 175 mg m^−2^) since two patients experienced febrile neutropenia (no hospital admission was required). Therefore, the recommended doses of the two drugs to be used in the phase II study were those of level B (EpiDx, 75 mg m^−2^; P, 175 mg m^−2^).

Nonhaematological toxicities were mild. Baseline median LVEF was 63.3% (range 57–70%) and no significant modification was observed after the sixth course of treatment in the whole patients' population.

Even if activity was not the end point of the phase I part of the study, considering the fact that all patients enrolled were chemotherapy naïve, responses were assessed, with the same criteria of phase II, every three courses of treatment. Twelve out of 13 patients were evaluable (one patient in group C was lost to follow-up after the first course of therapy). Six patients (50%) showed a complete response (two patients from group B and four patients from group C) and six patients showed a partial response; the best response was obtained after three courses of treatment.

### Phase II study

A total of 100 courses were administered with a median of 5 per patient (range 1–6). Myelosuppression, in particular neutropenia, was the most common toxicity (Table 3); grade 3–4 neutropenia was observed in 16 and 35% of the courses, respectively.

Seven episodes of febrile neutropenia were documented (30% of the patients) and one patient required hospital admission for parenteral antibiotic treatment. Patients having febrile neutropenia or grade 4 neutropenia (at any time in the trial) continued the treatment with paclitaxel reduced at 155 mg m^−2^ (72% of the cycles). No prophylactic GCSFs were administered. Other haematological toxicities were grade 3 thrombocytopenia in 2% and grade 4 in 1% of the courses; only one episode of grade 4 anaemia was reported. There were no treatment-related deaths. One patient received platelets and three patients received blood cell transfusion.

Nonhaematological toxicities were generally mild to moderate: only one episode of grade 3 diarrhoea was reported; grades 1 and 2 stomatitis occurred in two and three patients, respectively, grade 2 asthenia occurred in five patients and grade 2 vomiting in two patients. No clinically relevant neurotoxicity, cardiac dysfunction or hypersensitivity reactions were observed.

### Response and survival

In the first step six out of 18 evaluable patients obtained a complete response: the study proceeded to the second step in which one additional complete response was achieved (complete response rate 37% (95% confidence interval (CI)=16.3–61.4)) According to the statistical design, accrual was stopped at this point (20 total patients) as we had already obtained the figures we needed. Nine (47.3%) partial responses were achieved with an overall response rate of 84.2% ((95% CI=60.4–96.6)). Three patients progressed during chemotherapy. One patient was not evaluable for response (interruption of treatment after the first course because of arrhythmia). Five out of 20 patients had brain metastases; after three courses of chemotherapy four patients obtained an objective response of brain metastases (one patient obtained a complete response and three patients achieved a partial response), one patient progressed during chemotherapy.

Median time to progression was 6 months and median overall survival was 13 months.

## DISCUSSION

The treatment of ED-SCLC must be considered as palliative for the majority of individuals; although chemotherapy is able to achieve high remission rate and to improve tumour-related symptoms, very few patients may be cured with standard combination regimens (overall survival at 5 years is <5%). However, response to treatment, in particular, complete response, has been suggested as the major indicator of long-term survival (Paesmans *et al*, 2000). In ED patients the doublet PE produced complete response rates ranging from 4 to 23% (Evans *et al*, 1985; Roth *et al*, 1992; Pujol *et al*, 2001) and the triplets CDE (cyclophosphamide, doxorubicin and i.v. etoposide) and CAV yielded similar figures (Ettinger *et al*, 1990; Roth *et al*, 1992; Ardizzoni *et al*, 2002). The plateau reached by standard treatments stresses the need to test new drugs and/or new strategies in an attempt to improve the disappointing clinical results obtained so far.

Among the last generation drugs, paclitaxel is particularly noteworthy; this drug is highly active in various malignancies and its documented activity, in refractory patients, makes it especially interesting for testing in SCLC (Smit *et al*, 1998). The experience with paclitaxel, in the treatment of SCLC, is still limited but promising: available data from literature strongly suggest a high antitumour activity of paclitaxel-based regimens, with toxicity profiles which vary according to the different drugs used in combination. Two recent randomised phase III trials evaluated the role of this drug in the first-line treatment of SCLC. One trial compared carboplatin, etoposide and vincristine (standard arm) *vs* carboplatin, etoposide and paclitaxel in patients with LD and ED. The study showed that, on the whole, the risk of death was significantly higher for patients receiving standard therapy compared to those allocated to the experimental arm. However, the significant benefit in survival, provided by the experimental regimen was limited to patients with early stage of disease; no difference in median survival emerged for ED patients. It is important to note that this group achieved a higher complete response rate with the paclitaxel-containing regimen (13.7 *vs* 7.8%) (Reck *et al*, 2003) but the higher activity is probably insufficient to modify the efficacy results significantly. Consistent achievements were also reported in the second trial which evaluate the impact of adding paclitaxel to the standard PE doublet in ED patients. The triplet required the prophylactic use of G-CSF (Niell *et al*, 2005). The study showed a complete response rate of 10% for the doublet (standard arm) and of 16% for the paclitaxel-based triplet. No difference in survival emerged between the two arms of this study, but this could be partially due to the more severe toxicity (including toxic deaths) reported in experimental treatment; however, 42% of patients experiencing grade 5 toxicity were older than 70 years.

In the present study, we tested a new platinum-free triplet (paclitaxel, epirubicin, etoposide) obtained by substituting cyclophosphamide with paclitaxel in a standard CDE regimen (cyclophosphamide, epirubicin and etoposide) often used in our Institution. The phase I study indicated the optimal doses of the three drugs to be combined and used in the subsequent phase II study; this was performed in different institutions in order to verify the applicability of the regimen in everyday practice. The primary objective of the study was the percentage of complete response rate considering its potential prognostic significance.

The antitumour activity achieved with our combination was very high: the overall response rate was 84.2% (95% CI=60.4–96.6%) and the 37% (95% CI=16.3–61.4) complete response rate was really promising. It is worth noting that these percentages were obtained in patients with unfavourable prognostic features: 85% of our patients had two or more sites of metastases in addition to the primary tumour. As expected the most frequent toxic effect was myelosuppression with grade 3–4 neutropenia recorded in 51% of the courses and seven episodes of febrile neutropenia. Toxicity data seemed to be comparable with those reported with standard CDE combination (grades 3–4 neutropenia in 92% of the patients; febrile neutropenia in 24% of the patients) and slightly higher than those reported for the PE regimen (grade 3–4 neutropenia ranged between 38 and 66% of the patients; febrile neutropenia ranged between 6 and 13% of the patients) (Roth *et al*, 1992; Ardizzoni *et al*, 2002; Niell *et al*, 2005). However, it should be noted that in our study no prophylactic G-CSF was used. As a matter of fact, in the study by Niell *et al* (2005), the prophylactic introduction of G-CSF, in the paclitaxel-containing arm, made the toxicity profile of the triplet comparable to that of the standard PE.

Other haematological and nonhaematological toxicities, in our study, were mild to moderate and clinically irrelevant.

In conclusion, our regimen seemed to be feasible and very active for the treatment of ED-SCLC: the multicentre nature of the present trial, in which nonspecialised hospitals have also been involved, and its feasibility in an outpatient setting, make this regimen worthy of future comparative trials. The introduction of prophylactic G-CSF could further improve the haematological toxicity profile of our regimen allowing an easier comparison *vs* a standard PE regimen in a large randomised phase III trial.

## Figures and Tables

**Table 1:** Study design of phase I

**Dose level**	**EpiDx**	**P**	**E**	**No. pts**
A	60	155	100	3
B	75	175	100	3
C	90	175	100	7

Epirubicin (Epidx); paclitaxel (P); etoposide (E).

**Table 2:** Patients' characteristics

	**Phase I**	**Phase II**	**Total**
No. patients entered	13	20	33
Median age (years) (range)	65 (61–70)	61 (50–70)	62 (50–70)
			
*Gender*			
Male	10	18	28
Female	3	2	5
			
*ECOG PS*			
0	9	12	21
1	3	6	9
2	1	2	3
Baseline LVEF (median)	63.3% (57–70%)	58% (50–71%)	
			
*Dominant site of disease*			
Viscera	11	9	20
Soft tissue/bone	2	11	13
			
*Number of metastatic sites*			
1	4	3	7
⩾2	9	17	26

ECOG: Eastern Cooperative Oncology Group; PS: performance status.

**Table 3:** Grades 3–4 haematological toxicity for all cycles

	***n*=100**	
	**NCI grade %**	
**Cycles**	**3**	**4**
Neutropenia	15.9	35.2
Thrombocytopenia	2.2	1.1
Anaemia	—	1.1

Seven episodes of febrile neutropenia.
